# Protocol for a two-arm feasibility RCT to support postnatal maternal weight management and positive lifestyle behaviour in women from an ethnically diverse inner city population: the SWAN feasibility trial

**DOI:** 10.1186/s40814-019-0497-3

**Published:** 2019-10-23

**Authors:** Debra Bick, Cath Taylor, Amanda Avery, Vanita Bhavnani, Victoria Craig, Andy Healey, Nina Khazaezadeh, Sarah McMullen, Bimpe Oki, Eugene Oteng-Ntim, Sheila O’Connor, Lucilla Poston, Paul Seed, Sarah Roberts, Michael Ussher

**Affiliations:** 1 Warwick Clinical Trials Unit, Warwick Medical School, University of Warwick, Gibbet Hill, Coventry, CV4 7AL UK; 2 School of Health Sciences, University of Surrey, Guildford, UK; 3 School of Biosciences, University of Nottingham, Nottingham, UK; 4 NCT, London, UK; 5 Guy’s and St Thomas NHS Foundation Trust, London, UK; 6 Health Service and Population Research, King’s College London, London, UK; 7 Department of Public Health, Lambeth Council, Lambeth, London, UK; 8 Department of Women and Children’s Health, School of Life Course Sciences, Faculty of Life Sciences and Medicine, King’s College London, London, UK; 9 Division of Population Health and Education, St George’s University of London, London, UK

**Keywords:** Postpartum, Maternal health, Weight management, Health behaviour

## Abstract

**Introduction:**

A high BMI during and after pregnancy is linked to poor pregnancy outcomes and contributes to long-term maternal obesity, hypertension, and diabetes. Evidence of feasible, effective postnatal interventions is lacking. This randomised controlled trial will assess the feasibility of conducting a future definitive trial to determine effectiveness and cost-effectiveness of lifestyle information and access to Slimming World® (Alfreton, UK) groups for 12 weeks commencing from 8 to 16 weeks postnatally, in relation to supporting longer-term postnatal weight management in women in an ethnically diverse inner city population.

**Methods/analysis:**

Women will be recruited from one maternity unit in London. To be eligible, women will be overweight (BMI 25–29.9 kg/m^2^) or obese (BMI ≥ 30 kg/m^2^) as identified at their first antenatal contact, or have a normal BMI (18.5–24.9 kg/m^2^) at booking but gain excessive gestational weight as assessed at 36 weeks gestation. Women will be aged 18 and over, can speak and read English, expecting a single baby, and will not have accessed weight management groups in this pregnancy. Women will be randomly allocated to standard care plus lifestyle information and access to Slimming World® (Alfreton, UK) groups or standard care only. A sample of 130 women is required.

Feasibility trial objectives reflect those considered most important inform a decision about undertaking a definitive future trial. These include estimation of impact of lifestyle information and postnatal access to Slimming World® (Alfreton, UK) on maternal weight change between antenatal booking weight and weight at 12 months postbirth, recruitment rate and time to recruitment, retention rate, influence of lifestyle information and Slimming World® (Alfreton, UK) groups on weight management, diet, physical activity, breastfeeding, smoking cessation, alcohol intake, physical and mental health, infant health, and health-related quality of life 6 and 12 months postnatally. An embedded process evaluation will assess acceptability of study processes and procedures to women.

**Ethics/dissemination:**

London–Camberwell St Giles Research Ethics Committee, reference: 16/LO/1422. Outcomes will be disseminated in peer-reviewed journals and presentations at national and international conferences.

**Trial registration:**

Trial registration number: ISRCTN 39186148. Protocol version number: v7, 13 August 17. Trial sponsor: King’s College London.

## Strengths and limitations of this study

- Postnatal interventions may be more effective than antenatal interventions at supporting weight management among women with higher BMIs, but evidence is needed.
- This feasibility trial will assess if women with high BMIs at pregnancy commencement or have normal BMIs but gain excessive gestational weight would be prepared to be randomised to standard care plus Slimming World® (Alfreton, UK) groups offered from 8 to 16 weeks postnatally, or to standard care only.
- Women will only be recruited from one study site; however, the site provides maternity care to a population with wide diversity. The intervention is available UK-wide.
- Blinding of study participants and assessors is not possible.

## Introduction

At around 6 to 8 weeks postnatally, two thirds of women weigh more than their pre-pregnancy weight [1], with evidence that women who commence subsequent pregnancies overweight or obese have higher risk of adverse outcomes for themselves and/or for their infants. Furthermore, a high BMI before, during, and after pregnancy is linked to poor maternal health behaviours including smoking, lack of regular exercise, and not breastfeeding [2–4]. Maintaining a high BMI postnatally is an important predictor of maternal future weight gain and obesity, hypertension, diabetes, and degenerative joint disease [5–7], with infants of obese women at greater risk of higher BMI and blood pressure in childhood and young adulthood [4]. In the absence of evidence to support the benefit of pregnancy weight management interventions and positive lifestyle behaviours, postnatal interventions are viewed as increasingly important [8, 9] in terms of enhancing life-course health and preventing adverse outcomes in subsequent pregnancies, but knowledge of how best to support and treat overweight or obese individuals, including postnatal women, remains a challenge [10, 11]. The timing of commencement, recruitment approaches, and content of postnatal interventions are unclear [5], including how best to ensure that socio-economic influences which may impact on maternal health and lifestyle behaviours are considered. This is important, as a significantly greater proportion of women from areas of high social deprivation in the UK have weight management problems [12], findings supported with work from the USA where excessive pregnancy weight gain and failure to lose weight were found to be highly prevalent among young, low income, ethnic minority women in one large cohort study [13].

Although around half of all UK women of reproductive age are overweight or obese [14], UK public health guidance does not recommend dieting and weight loss during pregnancy due to concerns about impact on infant outcomes [15] despite lack of evidence [16], and there is no current UK guidance on recommended pregnancy weight gain. Pregnancy-only interventions have measured impact on adverse outcomes including gestational diabetes, large for gestational age infants and caesarean births [17–20]. UPBEAT, a UK-based multicentre trial of a behavioural intervention based on changing diet to foods with a lower glycaemic index and increasing physical activity, aimed to reduce risk of gestational diabetes and large for gestational age infants [21]; 1555 women with a mean BMI of 36.3 kg/m^2^ (SD 4.8) were recruited. No differences were found in incidence of outcomes of interest. Gestational diabetes was reported in 172 (26%) women in standard care compared with 160 (25%) in the intervention group (risk ratio 0.96, 95% CI 0.79–1.16; *p* = 0.68). Sixty-one (8%) of 751 babies in the standard care group were large for gestational age compared with 71 (9%) of 761 in the intervention group (1.15, 0.83–1.59; *p* = 0.40).

Recent systematic reviews of postnatal interventions have highlighted the potential importance of these, but reported gaps in evidence of ‘what works’ and the low quality of evidence. A Cochrane review of diet and/or exercise for postnatal weight reduction [22] in which 12 trials contributed data on 910 women found women who exercised did not lose significantly more weight than women in usual care groups (two trials, *n* = 53, mean difference − 0.10 kg, 95% CI − 1.90 to 1.71), but women who took part in a diet (one trial, *n* = 45, mean difference − 1.70 kg, 95% CI − 2.08 to − 0.132) or diet plus exercise programme (seven trials, *n* = 573, mean difference − 1.93 kg, 95% CI − 2.96 to − 0.89) lost significantly more weight than women in usual care groups. Trials included women who were obese, overweight, or gained excessive gestational weight, with recruitment from 3 weeks to 24 months postpartum. Intervention duration ranged from 10 to 24 weeks, with content often delivered as a ‘package’. Only one trial was from the UK. Despite considerable study heterogeneity, the authors suggested diet and exercise together rather than diet alone could help women to lose weight postnatally because the former could improve women’s cardiovascular fitness level and preserve fat-free mass.

van der Pligt and colleagues completed a systematic review of interventions to reduce postpartum weight retention across all BMI categories [5]. Studies were included if postpartum weight was a primary outcome, and diet and/or exercise and/or weight monitoring were intervention components. Interventions commenced from 11 days to 9 months postpartum and included counselling, individualised physical activity plans, healthy eating groups, and clinic visits. Of 11 studies included, 10 were RCTs, none from the UK. Seven reported a decrease in postpartum weight retention, six of which included diet and physical activity delivered by different health professionals. No study considered cost-effectiveness, with wide heterogeneity in intervention implementation. Nevertheless, findings suggested that postnatal weight loss was achievable, although evidence of optimal setting, implementation, intervention duration, and recruitment approach was unclear.

The potential for postnatal interventions to impact on other maternal health behaviours was considered in a review by Hoedjes et al. [23] which also reported poor-quality evidence. Eight of 17 included studies assessed effects on weight loss, and 9 on smoking cessation and relapse prevention. Of the weight loss studies, five reported significant effects of combined diet and exercise. Two of the four studies which assessed smoking relapse prevention found no evidence of effect. Four studies included interventions for smoking prevention and prevention of relapse. Although authors recommended that existing postpartum lifestyle interventions could support weight loss and smoking cessation or prevent smoking relapse, caution is needed. There was wide variability in study methods, and details of study selection, data extraction processes, and assessment of study quality were not provided.

The potential for the postnatal period to be a more appropriate time to consider support for weight management and positive lifestyle behaviours is clear, yet high-quality evidence of effective interventions is lacking. Based on evidence from general population studies that commercial organisations may be more effective at supporting weight management than healthcare providers [24], this trial is being undertaken to assess the feasibility of conducting a future definitive RCT to determine effectiveness and cost-effectiveness of lifestyle information and access to Slimming World® (Alfreton, UK) groups for a 12-week period, from 8 to 16 weeks postnatally, on long-term postnatal weight management and positive lifestyle behaviour among women from an ethnically diverse inner city population.

## Objectives

The following are the specific trial feasibility and trial process objectives to inform if a future definitive trial could be undertaken. Findings will inform if postnatal women would be prepared to enter a study of weight management, when would be an optimal time to intervene, content of a pragmatic and accessible intervention, if an intervention could impact on other health areas, and outcomes likely to be of most importance in a future trial.

### Trial feasibility objectives

The trial feasibility objectives are as follows:

- To assess recruitment and retention rates
- To estimate the effect size for a likely primary study outcome in a future definitive trial, namely difference between study groups in weight change from booking to 12 months postbirth, expressed as percentage of weight change or weight loss in kilogrammes
- To estimate impact of lifestyle information and access to Slimming World® (Alfreton, UK) groups on maternal weight change from first antenatal visit to 12 months postnatally
- To explore influence of lifestyle information and access to Slimming World® (Alfreton, UK) groups including weight management, diet, physical activity, breastfeeding, smoking cessation, alcohol intake, physical and mental health, infant health, sleep patterns, body image, self-esteem, and health-related quality of life at 6 and 12 months postnatally
- To assess resource impacts across different agencies likely to be of relevance and identify data appropriate for economic evaluation in a definitive RCT
- To decide if criteria to inform progression to a definitive RCT have been met

### Trial process evaluation objectives

The trial process evaluation objectives are as follows:

- To assess acceptability of trial procedures and intervention
- To assess variation in Slimming World® (Alfreton, UK) groups attended by women
- To assess sources of weight management support accessed by women (in particular assess risk of contamination) (Table 1)

**Table 1:** Study objectives, outcomes, criteria for success, and method of analysis

Objectives	Feasibility outcomes (trial)	Criteria for success	Method of analysis
Maternal weight change (proposed primary endpoint for future definitive RCT)	Difference in percentage of maternal weight change between trial groups from antenatal booking to 12 months postnatally	Percentage of weight change/weight loss in kilogrammes	Estimated differences/95% CI calculated (significance at 5%) Pre-planned sub-group analysis by BMI category
Recruitment and retention	Uptake/time to recruit 190 women from BMI categories of interest	Complete recruitment within 6 months	Descriptive
	Loss to follow-up under 30% at 12 months	Retain 130 women to 12 months	Descriptive
Explore influence of lifestyle information and Slimming World on lifestyle/health behaviours	Dietary intake, EPDS score, breastfeeding uptake and duration, sleep patterns, smoking, alcohol consumption, self-esteem, infant health, body image	High completion of all included measures at each follow-up point	*N* (%) for binary and categorical variables and mean (SD), or medians/geometric means for continuous variables
Assess feasibility of collecting resource utilisation and cost data	Completion of EQ-5D-5L, Adult Service Use Schedule, relevant data from women’s maternity records	High completion of all included measures at each follow-up point	Multivariate/sensitivity analysis
Objectives	Outcomes (process)	Criteria for success	Method of analysis
Acceptability of trial process procedures	Reasons for taking part/dropping out, expectations, understanding of study aims, attendance at follow-up appointments, acceptability of surveys	Processes and procedures acceptable, high completion of follow-up measures	Framework method, descriptive
Acceptability of intervention	Women commence and complete Slimming World sessions offered	Depth of understanding of barriers and facilitators to uptake and retention at sessions, so this can be maximised in a future trial	Framework method, descriptive
Variation in Slimming World groups attended by women by date/time of day	Range of variation in Slimming World groups attended	Availability of data from Slimming World	Descriptive
Sources of weight management support control group accessed/additional support intervention women accessed	Extent and type of weight management support used by control women, additional support used by intervention women	Risk of contamination re access to commercial weight management support by control group	Descriptive

## Methods and analysis

This protocol paper has been written following the SPIRIT and TIDieR guidance [25, 26].

### Study design

This is a single centre, randomised two-arm feasibility trial, with a nested mixed-methods process evaluation.

### Setting

The study will be conducted in one maternity unit in inner city London, serving an ethnically diverse population and providing maternity care for over 6000 women annually.

### Target population

The target populations are women who are overweight (BMI 25–29.9 kg/m^2^) or obese (BMI ≥ 30 kg/m^2^) as identified at their first antenatal contact and women with a normal BMI (18.5–24.9 kg/m^2^) at pregnancy booking who gain excessive gestational weight as assessed at 36 weeks gestation [27]. Women will be eligible for recruitment if they meet the following criteria:

- Aged 18 and over
- Speak and read English
- Expecting a single baby
- Have not accessed weight management groups during index pregnancy

#### Exclusion criteria

Women will be excluded if they meet the following criteria:

- < 18 years old
- Insufficient understanding of spoken and written English
- Current diagnosis of major psychiatric disorder
- Foetus has known abnormality
- Involvement in another postnatal study to reduce ‘burden’ of research participation
- Identified medical complications (for example, cardiac disease, type 1 diabetes)
- Identified eating disorders
- Previous surgery for weight management

### Recruitment

Women with BMIs ≥ 25 will be identified from their antenatal booking details. At approximately 26 weeks gestation, identified women in this group will receive a study letter which briefly explains study aims and advises that a research midwife will be in contact within 2 weeks to explain the study further. The letter will also explain how the woman can contact researchers if she does not want to receive further details. The research midwife will call women who have not asked to be removed from the contact list.

Women with normal BMIs at pregnancy commencement, but gain excess pregnancy weight as assessed at around 36 weeks gestation against IoM criteria [27], will be offered the opportunity to self-refer to the research midwives, through advertising at the study site and midwifery and obstetric referrals (from around 28 weeks gestation). Women will be asked to contact the research midwives to arrange to be weighed at around 36 weeks gestation. If women in this group do not respond to study advertising within 3 months of commencement of recruitment, a similar opt-out approach for women with high BMIs will be adopted, following relevant ethics amendments. An opt-out letter would be sent to all women with normal BMI at booking at around 32–34 weeks gestation to explore feasibility of this approach.

All women identified will be offered a patient information leaflet by research midwives prior to seeking consent at around 36 weeks gestation. Women interested in participating will be met by research midwives at the study site to obtain consent and complete the baseline questionnaire. Women who gain excessive gestational weight could be recruited at an antenatal appointment if this takes place around 36 weeks gestation.

All women who are eligible, recruited, and provide written consent will be randomised to either of the following:

- Standard care plus lifestyle information and postnatal access to Slimming World® (Alfreton, UK) weight management groups
- Standard care only

A screening form will be completed by the research midwife to record women approached, their eligibility, and at what point women may have declined to take part (when first approached or at consent) and reasons for this, if women are willing to provide information.

A separate patient information leaflet will be mailed to recruited women purposively selected from both trial groups and invited to participate in a telephone or face to face interview at 12 months postnatally. Similarly, women allocated to the intervention who are purposively selected for interview about their experiences on completion of their Slimming World® (Alfreton, UK) groups will be sent a separate study information leaflet to seek consent to participate in this stage of the study process evaluation.

### Treatment allocation and randomisation

Randomisation and allocation will be carried out by KCL’s Clinical Trials Unit web-based system. Women will be registered on the InferMed MACRO web-based data entry system by a research midwife prior to randomisation to allocate each a unique study number ‘PIN’. Research midwives will access the system and, using the PIN, initials, and date of birth, request randomisation. Unit of randomisation will be individual participant, allocated in a ratio of 1:1 to intervention and control. Selection bias will be minimised by ensuring all women eligible and recruited have equal opportunity of being allocated to each study group and follow-up completed, with information on women randomised and allocated but who opt out presented but clearly indicated as such. Use of intention to treat (ITT) analysis will limit attrition and analytical bias.

### Blinding

It will not be possible to ‘blind’ research midwives or women to allocation. Those responsible for analysis will be blinded to allocation.

### Interventions

**Fig. 1:**
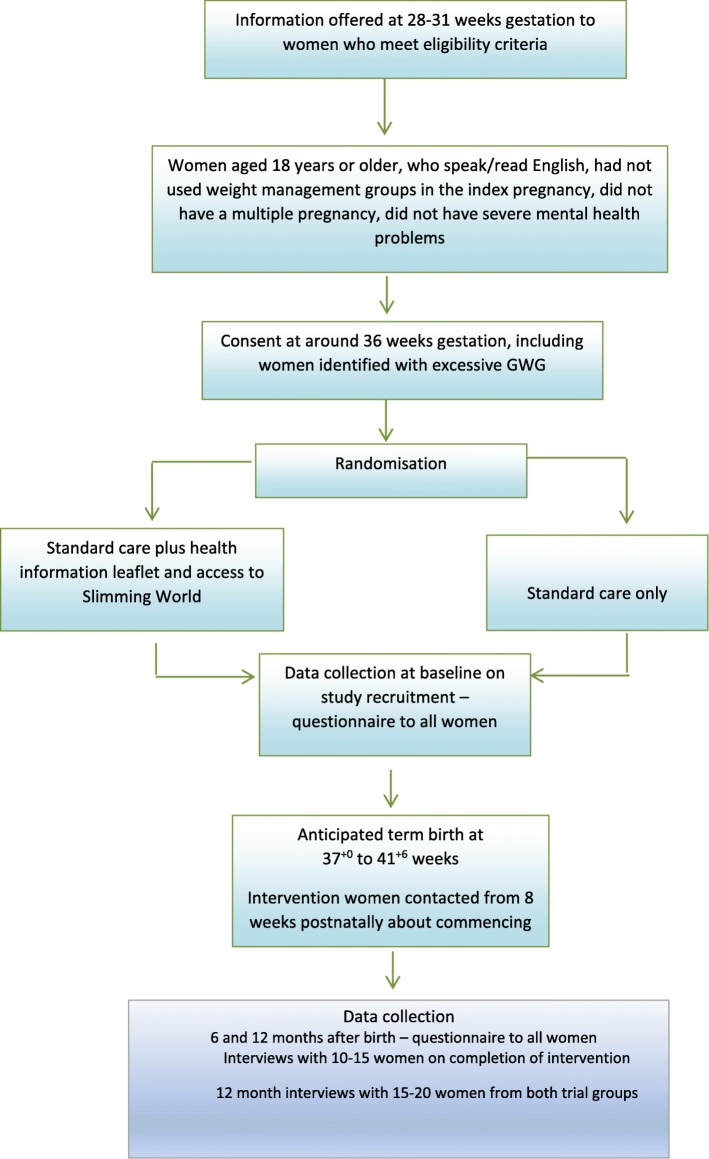
CONSORT study flow diagram for supporting women with postnatal weight management (SWAN) feasibility trial

Standard care (described below), plus information on positive lifestyle behaviours from late pregnancy and access to a 12-week weight management group (Slimming World® (Alfreton, UK)) commencing from 8 weeks up to 16 weeks postnatal (see Fig. 1 flow chart).

An evidence-based positive lifestyle leaflet reflecting current NICE guidance on breastfeeding, diet, importance of smoking cessation/prevention of relapse, reducing alcohol, and managing sleep [10, 28] will be offered to women allocated to the intervention.

Slimming World® (Alfreton, UK) content is underpinned by group behaviour change techniques targeted to support individuals make healthier food choices and increase physical activity (www.Slimmingworld.co.uk). Groups are run using a standardised approach, with in-built quality assessment procedures [7]. Behaviour change techniques are supported by social cognitive theory, with a focus on motivation and self-efficacy for weight management and reducing relapse, including goal setting, self-monitoring, social support, and positive reinforcement [29, 30].

Consultants who lead groups receive standardised training overseen by Slimming World® (Alfreton, UK) dietitians and nutritionists which includes motivational techniques to support positive lifestyle changes to manage weight, nutrition, food facts, and role of exercise and activity in health and weight management. Groups follow a standard format, starting with a weigh in, followed by discussion of group member’s experiences of weight management to help change habits, share healthy swaps, and discuss what to eat. Sessions can include basic cooking skills, taking cost, cultural preferences, and time constraints into account.

A food optimising system encourages adherence to healthy eating, and physical activity encouragement includes facilitation of behaviour change, redefining what ‘activity’ can include. Slimming World® (Alfreton, UK) will record initial and ongoing attendance and weekly weight. Women can attend for up to 12 groups at no-cost, which run over 14 consecutive weeks to allow 2 ‘holiday’ weeks within offer. After this time, women can choose to continue and pay the standard weekly fee.

A research midwife will contact women at 8 weeks postnatally to provide a dedicated Slimming World® (Alfreton, UK) telephone number for member services. A Slimming World® (Alfreton, UK) consultant will provide the woman with information on the local groups she can join, times, and venue (standard practice). Women can choose which group they attend and when they commence groups within the 8 to 16 week ‘window’ to fit with their health, lifestyle, and family demands. Women can take their babies with them.

We will assess adherence to allocation protocols, loss to follow-up, and women’s views of acceptability and sustainability. To capture data on whether women attend for each complete Slimming World® (Alfreton, UK) group session or leave after the initial weigh-in, we will ask women to complete in their follow-up questionnaires how many groups they attended and stayed for the whole session. Follow-up will capture data on women who continue attending groups or achieve their goal and stop attending to compare outcomes of interest with the control group.

Slimming World’s® (Alfreton, UK) quality assurance procedures ensure standardisation of weight management groups, including weekly calls between group consultants and their team managers. As consultants are from the local area, they will be aware of cultural preferences, time, and budget constraints women face and can advise on basic cooking skills and local shops selling healthy foods within women’s budgets. The Food Optimising system used in Slimming World® (Alfreton, UK) groups includes pictorial use of recipes for individuals who may not have a high level of understanding of written English. As our postnatal population is geographically mobile [31], women who move from the area can transfer their group membership to another local group anywhere in the UK.

Women from both trial groups will be asked to attend an appointment with a research midwife to be weighed either at the study site or at their home at 6 and 12 months. Travel costs and £10 Love2Shop voucher to thank women for their time will be offered.

### Standard care

Women allocated to standard care only will receive standard NHS maternity care to 8 weeks postnatal. This could include, for example, routine midwifery and health visitor contacts for infant feeding assessment and assessment of birth recovery. Women will be offered a routine contact with their GP at around 6–8 weeks postnatally. We will ask all recruited women at their 6 and 12 month follow-up about their experiences of using weight management groups or other sources of support for weight management, healthy lifestyle, and activity.

### Trial objectives

Feasibility objectives reflect MRC guidelines for complex interventions [32] with important exceptions. The aim is not to evaluate the intervention itself. Slimming World® (Alfreton, UK) groups are ‘standardised’, with robust mechanisms to ensure intervention fidelity. Our process evaluation is not designed to answer some questions seen in complex evaluations regarding generalizability of the intervention, assurance that implementation/delivery of the intervention is consistent across sites, or determine mechanisms of impact. This study reflects a pragmatic trial approach—evaluating the impact of an intervention where women can choose which group to attend and can switch groups, exactly as if they were a ‘standard’ self-referred member of Slimming World® (Alfreton, UK).

### Trial feasibility objectives

The primary feasibility objective is to estimate the effect size for a likely primary study outcome in a future definitive trial, namely difference between study groups in weight change from booking to 12 months postbirth, expressed as percentage of weight change or weight loss in kilogrammes. Mean percentage change across all women gives greater power, and fewer women will need to be recruited. We will undertake pre-planned sub-group analysis of the primary assessment in women of different booking BMI categories. The primary endpoint for a future trial will be selected on grounds of power.

Other objectives include those most appropriate to support meeting the primary objective and inform progress to a definitive RCT. These will include rates of 5% and 10% weight reduction and changes in relation to aspects of healthy lifestyle and health behaviours, including diet and nutrition, breastfeeding, physical activity, smoking cessation, alcohol intake, self-esteem, and body image. Some included measures have been validated in relevant populations, including among women of reproductive age and women who have recently given birth.

Measures will be included in questionnaires at 6 and 12 months postnatally. Those marked with ‘**’ will also be included in the baseline questionnaire. Questionnaires will be ‘tailored’ for the intervention or standard care arm, to enable questions on uptake of support for weight management to be included at 6 and 12 months to inform trial process outcomes:

- Dietary intake: The Dietary Instrument for Nutritional Education (DINE© University of Oxford)** [33]
- Soft drink intake: questions developed for the study
- Physical activity: The International Physical Activity Short-Form** [34]
- Mental health: Edinburgh Postnatal Depression Scale [35]
- Breastfeeding intent**, uptake, and duration, questions developed for study
- Sleep patterns: questions developed for the study
- Smoking: smoking status/cigarette dependence** [36]
- Alcohol consumption: Alcohol Use Disorders Identification Test** [37]
- Self-esteem: Rosenberg Self-Esteem Scale** [38]
- Infant health: questions developed for study
- Impact on body image [39]
- Resource utilisation and costs outcome measures: the EQ-5D-5L** and the Adult Service Use Schedule** [40]

### Trial process evaluation objectives

The process evaluation will focus on the following key aspects:

- The acceptability of study processes and procedures
- The acceptability of the intervention and how it is experienced by women (those who complete at least 10/12 Slimming World® (Alfreton, UK) groups and those who do not; those who complete each group attended for the full session or leave after being weighed; those who continue to attend Slimming World® (Alfreton, UK) groups on completing the intervention and those who do not)
- The likely variation in groups attended by women in relation to date/time of day, and whether women stay with one consultant/group or try different groups
- Sources of weight management support control group women may have accessed; additional sources of weight management support intervention group women may have accessed

Data will be collected using questionnaires and semi-structured interviews with a sub-sample of women. Questionnaires at 6 and 12 months include sections designed to capture information on weight management views and use of strategies among all women, and on Slimming World® (Alfreton, UK) attendance, barriers, and facilitators among intervention women only.

Semi-structured qualitative interviews will be conducted at two time points with intervention women (*n* = approx. 10 women at 6 months; 15–20 at 12 months) to explore the acceptability and sustainability of the intervention, and acceptability of trial procedures. This will include targeting women who did and did not join Slimming World® (Alfreton, UK), those who did/did not attend the 12 sessions offered, and those who did/did not lose weight. At 12 months, a sub-sample of control group women will also be interviewed to explore study processes and experiences of participating, including reasons for taking part/dropping out, recruitment and randomisation (expectations/understanding of the study aims), views on outcome measures, attendance for weighing appointments as part of study follow-up, and lifestyle behaviours. Topic guides for both groups will be informed by the COM-B model of behaviour change [41], exploring barriers and facilitators to weight management/the intervention according to capabilities, opportunities, and motivations. Control group women will be asked about the impact, if any, of participating on their levels of physical activity or any dietary changes.

Telephone or face to face interviews will be offered. Sampling will be based on maximum diversity in relation to age, parity, ethnicity, and socio-economic status and reflect the range of weight loss/gain of the sample.

### Health economics

The health economic component will review quality and completeness of economic data generated by the trial itself (e.g. women’s self-report data on service contacts and quality of life outcomes, data from women’s maternity records) and data from external sources (e.g. unit cost data for costing service contacts and estimating intervention costs) necessary for conducting an economic evaluation in a future definitive trial. An assessment will also be undertaken of suitability of existing economic models that could support additional modelling of longer-term (out of trial) resource impacts and health outcomes linked to observed (within trial) impacts on weight loss as part of a definitive trial. This will identify whether or what additional evidence and modelling would need to be undertaken to support evaluation of longer-term costs and outcomes.

The health economic component will include primary analysis of economic data generated by the trial to gain preliminary insight into intervention cost-effectiveness. This will include the following:

- Analysis of differences in overall costs between trial groups, including service contacts, clinical resource use, and weight management intervention costs. Sub-group effects on differences in costs between groups will be explored with specific reference to baseline BMI. This, with evidence on sub-group analysis of weight loss outcomes, will inform whether a definitive trial should be designed to evaluate variability in intervention cost-effectiveness between relevant sub-groups.
- A preliminary within trial cost-effectiveness analysis of the weight management intervention versus standard care using comparative feasibility trial data on resource use and costs and quality of life outcomes over a 12-month follow-up.

### Sample size calculation, selection, and loss to follow-up

A sample size of 190 women will allow a 30% loss to follow-up to ensure we achieve our required sample size of 130 women. This study is designed firstly to establish the rates at which women could be recruited and retained in a future definitive RCT and estimate critical parameters with necessary precision to inform sample size requirements. In particular, we require estimates of the standard deviation, and design effect for the primary endpoint, allowing for clustering by intervention group. One hundred thirty women will allow estimates of the required sample size for any given clinically important difference to within 30% of the true value.

Based on published data [17, 42] the mean (SD) percentage weight change following a Slimming World® (Alfreton, UK) programme of 12 weekly groups is − 5.5% (3.3). Assuming these numbers are typical, 65 women in each group (130 in all) would be required to detect a difference of 2% between active and control groups with 90% power. Of around 6600 women who give birth at the reference maternity unit over 12 months in 2017, 40% were overweight, 15% of whom were obese. Data on women with excessive GWG are not routinely collated. Potentially, 55 women booking each week would meet obese/overweight inclusion criteria. Recruiting 7–8 women each week over an 8-month (32 week) period would be sufficient to achieve the desired sample size to meet the aims of this feasibility study.

Compliance issues are an important consideration. Loss to follow-up among postnatal women can range from 30 to 40% [43, 44]. Experience of research in similar populations has shown that planned initiatives including flexible follow-up appointments, travel expenses, vouchers for returning questionnaires, and sending of reminders can reduce loss to follow-up. Follow-up appointments will be offered at weekends and week days, with the option to complete questionnaires at these appointments.

### Analysis

Process data will be analysed separately before examining relationships between quantitative and qualitative data, with synthesis completed in line with O’Cathain et al. [45]. Quantitative data will be entered onto the MedSciNet web-based data entry system. Retention and adherence will be considered from recruitment rate, consent rate, withdrawal and loss to follow-up (with reason), departures from randomised treatment, and prevalence of SAEs reported by treatment group and overall. Estimated differences and 95% confidence intervals will be calculated for specified primary and secondary analyses (significance at 5%). Sensitivity analyses will assess robustness of conclusions to missing outcome data and departures from randomised treatment. Analyses of potential efficacy will be based on the ITT sample, utilising follow-up data from all randomised women.

Differences between arms will be compared at 6 and 12 months postbirth adjusting for important prognostic factors (parity, maternal age, ethnicity, BMI at booking, and (as appropriate) baseline measurement). Data to inform process outcomes will be evaluated using descriptive analysis. Numbers (with percentages) will be presented for binary and categorical variables and means (standard deviations), or medians (with lower and upper quartiles) or geometric means for continuous variables will be presented. Stata version 15.1 or later will be used for analyses (StataCorp, College Station, TX).

Interviews will be recorded with women’s permission and transcribed and analysed using the framework method for thematic analysis [46]. Analyses will be deductively informed by the COM-B model for behaviour change [41]. Themes that emerge inductively will be captured, and efforts made to identify and explore disconfirming/outlier cases**.** Key topics and issues will be identified through familiarisation with transcripts by two researchers who will initially work independently and then together to discuss and agree the final coding framework with a third researcher. A series of thematic charts will be developed according to the coding framework, and data from each transcript summarised under each theme, enabling examination of similarities and differences of views within and between transcripts, and use of a constant comparative approach. Quantitative and qualitative data on acceptability of the intervention and other aspects of feasibility will be integrated using mixed-methods matrices [46].

### Economic analysis

Economic analyses will be conducted from an NHS/personal social services perspective on the assumption that if the intervention were commissioned for the target population, full costs would be met from NHS or local authority public health resources. A review of evidence will be completed to assess if the wider evidence base would support economic modelling of impacts in a future study.

Data on self-reported service use for specific service items will be collected using a modified version of the Adult Service Use Schedule (AD-SUS). Service use for individual items between intervention and control groups will be compared descriptively (means, range, and standard deviations). Service contacts will be costed using existing published unit cost data. Trial participants allocated to the intervention will be allocated an intervention cost based on prices charged by the provider organisation. Total costs from baseline to completion of follow-up (12 months) will be calculated for each participant as the sum of the cost of all service contacts plus intervention costs (for the intervention group).

Differences in mean total costs between trial groups will be evaluated through multivariate analysis controlling for baseline covariates. Interaction effects between the treatment allocation variable and variables identifying baseline BMI groupings will be used to test for sub-group effects. The base-case analysis will evaluate the mean cost difference on a complete case basis and based on intention to treat (ITT).

EQ-5D-5L data at baseline and follow-up will be used to evaluate quality of life outcomes in terms of a quality adjusted life year (QALY) equivalent over the 12-month follow-up period. Multivariate analysis will evaluate the mean difference in QALY outcomes between trial groups adjusting differences in baseline covariates. Mean QALY differences will be combined with mean cost differences to evaluate cost-effectiveness on a complete case and ITT basis. Sampling uncertainty will be modelled using a simulated non-parametric bootstrap distribution of cost and QALY difference pairings within the cost-effectiveness plane and through subsequent generation of a cost-effectiveness acceptability curve (CEAC).

Sensitivity analysis will include the effects of missing data on base-case conclusions and sensitivity of estimated mean cost differences to alternative multivariate modelling specifications, and sensitivity to departures to randomised treatment.

### Monitoring

The trial will be supervised by an independent Trial Steering Committee (TSC). An independent data monitoring committee (DMC) is not required to oversee the safety of subjects in the trial. As this is not a clinical trial of an investigational medical product, the TSC will take overall responsibility for trial conduct.

### Data management

Quantitative data will be collected using specific study data collection forms, and processed and monitored centrally for consistency, viability, and quality by the Core Project Team. Data will be screened for out-of-range data, cross-checked for conflicting data within and between collection forms using computerised logic checking screens, and processed using a double data-entry system by an independent data clerk. The trial statistician will monitor data for consistency, viability, and quality using a bespoke data management system (MedSciNet Ltd., Stockholm, Sweden). The MedSciNet programmer will run trial-specific programmes to extract certain fields from the database (as requested by the chief investigator or trial statistician), cross-check certain information, and, with the chief investigator, review results generated for logic and any patterns or problems.

### Patient and public involvement

The original research questions, study design, intervention, and outcomes were developed with the support of six local women who had commenced pregnancy with higher BMIs and had experienced support of weight management both during and after pregnancy. To support the study as it progresses, four of these women will form an Expert Patient Group which will meet three times a year to discuss study progress, overcoming any barriers to recruitment, content and completion of baseline and follow-up questionnaires, interview schedules, access and take up of the intervention, and implications of findings for a future definitive RCT. All women recruited will be offered the opportunity to receive a summary report of the trial.

### Confidentiality

All data from maternity records, questionnaires, interviews, and Slimming World® (Alfreton, UK) on women’s attendance and weight management progress will be anonymised, kept confidential, and managed in accordance with the Data Protection Acts 1998 and 2018, NHS Caldicott Guardian, the Research Governance Framework for Health and Social Care, and Research Ethics Committee Approval. Each woman will be allocated a unique study reference number. All records will be kept in a secure storage area with limited access. Clinical information will not be released without the written permission of the participant, except as necessary for monitoring and auditing by the sponsor, its designee, regularity authorities, or the REC. No patient identifiable data will be used in any publications or presentations relating to this study.

### Ethics and dissemination

Study findings will be published in high impact journals, in line with the CONSORT guidance [47]. Our expert PPI group will advise on dissemination of findings relevant for women and their families, in line with the INVOLVE guidance. Slimming World® (Alfreton, UK) will have no access to trial data or be involved in any aspect of trial analysis or interpretation of trial results. The funders will have no role in trial analysis or interpretation of trial results.

## Discussion

This feasibility trial is designed to provide robust data on whether it is possible to undertake a future definitive RCT of a postnatal weight management and lifestyle behaviour intervention among women from an ethnically diverse inner city setting. Support for weight management is important, given the longer-term health impacts of a higher BMI at pregnancy commencement, or as a consequence of gaining EGWG, on women and their children, but evidence of how best to support these women is lacking. Prior to undertaking a definitive RCT of effectiveness, evidence is needed to see if such a trial could be undertaken, if postnatal women would be prepared to enter a study of weight management, when would be an appropriate time to offer such an intervention, if a weight management intervention could impact on other positive health behaviours, confirm outcomes likely to be of most importance in a future trial, and if criteria to proceed to a definitive trial have been met.

## Data Availability

Not applicable.
